# Psychoeducational preventive treatment for women at risk of postpartum depression: study protocol for a randomized controlled trial, PROGEA

**DOI:** 10.1186/s12888-016-1162-5

**Published:** 2017-01-13

**Authors:** Amaia Ugarte Ugarte, Purificación López-Peña, Carmen Serrulla Vangeneberg, Julia Gemma Torregaray Royo, Maria Asunción Arrieta Ugarte, Maria Teresa Zabalza Compains, Maria Pilar Riaño Medrano, Nerea Muñoz Toyos, Edurne Arenaza Lamo, Maria Begoña Beneitez Dueñas, Ana González-Pinto

**Affiliations:** 1 Department of Psychiatry, University Hospital of Alava-Santiago, Vitoria, Spain; 2 Centre for Biomedical Research Network on Mental Health (CIBERSAM), Madrid, Spain; 3 School of Medicine, University of the Basque Country, Vitoria, Spain; 4 Obstetrics and Gynecology Service, OSAKIDETZA, Basque Country, Spain

**Keywords:** Prevention and Control, Depression, Postpartum, Cognitive Therapy, Depression, Quality Of Life, Motor Activity

## Abstract

**Background:**

Postpartum depression is a disease with a prevalence of 20% that has deleterious consequences not only for the mother but also for the baby and can cause delays in physical, social and cognitive development. In this context, the European Union Committee on Public Health has declared it essential that preventative measures are taken by centres providing care for women with a multidisciplinary approach. PROGEA is a multicentre, single-blind randomized, 3-year, longitudinal clinical trial aiming to evaluate the efficacy of a psychoeducational programme in preventing postpartum depression in at-risk women, based on a range of clinical variables, and explore prognostic factors. This paper describes the methods and rationale behind the study.

**Methods:**

We will study women receiving treatment as usual plus a psychoeducation cognitive behavioural therapy (CBT)-based intervention and a control group receiving only treatment as usual. The sample will be recruited from an incidental sampling of pregnant women in two health regions. We will recruit 600 women in the third trimester of pregnancy who consent to take part in the study. Almost half of the women, about 280, would be expected to have some risk factors for postpartum depression. All those found to have risk factors will be evaluated, and we estimate that a quarter will be classified as at-risk of developing postpartum depression as measured with the Edinburgh Postnatal Depression Scale. This subset will be randomly allocated to receive treatment as usual with or without the CBT intervention. Six sessions of CBT (1 individual and 5 group) will be offered by a psychologist.

**Discussion:**

Findings from this study will be used to design a definitive study that will examine the clinical and cost-effectiveness of the CBT-based intervention in improving the mood of women in the postpartum period.

**Trial Registration:**

ClinicalTrials.gov Identifier: NCT02323152; Date: December 2014.

## Background

Treatment of major depression is an objective of the European Union and in recent years the World Health Organization (WHO) has identified improving maternal mental health as a fundamental part of the Millennium Development Goals [1]. Today, peripartum depression (as defined in DSM-V) [2] is attracting greater attention from clinicians and researchers, as a condition that affects not only the mother, but also the health and development of the child health [3–5]; for these reasons, its prevention is particularly important [6].

The most frequent psychopathological disorders in women during pregnancy and the puerperium are depression, stress and anxiety [7]. Postpartum depression (PPD), in particular, is one of the most common complications in this period, affecting nearly 20% of all women after childbirth each year [8], but many cases go undetected and there is no international consensus on the best screening method [9].

PPD is defined as development of a major depressive episode with onset during pregnancy or within 4 weeks after childbirth and in which depressive symptoms are present for at least 2 weeks [2]. The risk factors for developing PPD include biological variables such as hormonal changes that women experience during pregnancy and after delivery; these are necessary to ensure their health during this period and are part of preparing the body for both childbirth and breastfeeding, but can cause depression in a subgroup of vulnerable women [10]. In addition to biological factors, there are psychosocial factors, among which we should highlight a personal or family history of depression or PPD and having experienced mood depression or anxiety during pregnancy [11].

Specifically, rates of PPD of more than 70% have been found in women with previous depressive symptoms [12]. Other risk factors described include: personal vulnerability of women [13], the temperament of the child [14], poor family and social support during the postpartum period [6, 15], relationship problems during pregnancy [16, 17], traumatic experiences in previous pregnancies [18] or low socioeconomic status [19].

Psychological therapy for depression has shown to be an effective treatment in depressed patients of all ages and all degrees of severity, including PPD [20]. Given the consequences of the disease, another strategy would be to administer preventive treatment with a lasting effect that could protect women against developing this type of depression. A systematic review found that 13/17 studies with successful psychological treatment targeted an at-risk population, and 4/7 trials using interpersonal therapy demonstrated success of the intervention versus a control condition [8].

The aim of this study was to identify women at risk of PPD using a protocol [21], investigate the efficacy of a structured programme of psychoeducational therapy designed ad hoc in preventing the development of PPD in at-risk women and explore prognostic factors for this type of depression.

## Methods/Design

We registered this single-blind randomized clinical trial in 2014 (identifier NCT02323152). Women will be recruited in the third trimester of pregnancy from 2015 to the completion of the study. Women that agree to participate and sign the informed consent form will then be assessed through a first screening visit by their reference midwife; specifically, the midwife will record sociodemographic data and the presence of various previously described risk factors, including the personality vulnerability of the woman and her socioeconomic status.

Women with at least one risk factor will progress to the next phase of the study and will be assessed between 8 and 15 days after the birth of their baby, at the Araba University Hospital. Clinical data will be collected relating to the delivery. We will also evaluate the women’s mood with the Edinburgh Postnatal Depression Scale (EPDS) [22], the child’s temperament with the Merrill-Palmer-Revised Scales of Development [23] and the current and overall physical activity of women with the International Physical Activity Questionnaire (IPAQ) [24] and Global Physical Activity Questionnaire (GPAQ) [25] using the Spanish versions of these tools. A score between 7.5 and 12.5 on the EPDS indicates depression risk and women with scores in this range will progress to the next phase of the study. In this phase, the participants will be randomized with Random Allocation Software 1:1 to the experimental or control group (treatment as usual). Women in the experimental group will receive six (1 individual and 5 group) sessions of the psychoeducational intervention and their status will be evaluated weekly. The control group will continue to have regular appointments with the midwife and gynaecologist and will be assessed again 6 weeks after inclusion in the trial.

We will recruit patients from five health centres in Vitoria and two health centres in Donostia, and the study is being coordinated by the Psychiatry Research team of Araba University Hospital, which is a member of the Center for Biomedical Research Mental Health Network (CIBERSAM). Patients meeting all the inclusion criteria and none of the exclusion criteria (listed below) will be included in the study.

The trial will be conducted in accordance with good clinical research practice, enabling the research to be carried out with low risk of bias and a high degree of external validity.

### Inclusion criteria

1. General criteria for inclusion in the 1st phase of the study:
  - Pregnant women in their third trimester. In the case of women under 18, inclusion will be notified to the Finance Ministry
  - Written informed consent of the participant. In the case of women under 18, their legal representative also must sign the document.
  - Inclusion criteria for 2nd phase of the study:
    - Presence of one or more of the following risk factors for developing PPD, assessed in the 3rd trimester of pregnancy:
      Depressive or anxious symptoms during pregnancy
      Personal history of severe mental disorder (schizophrenia and other psychoses, bipolar disorder, depressive disorder)
      Family history of severe mental disorder
      Concomitant medical diseases associated with depression (diabetes, heart disease, hypertension, obesity)
      Low or very low socioeconomic status
      Lack of support (partner, family, friends or others).
      b) A score ≥7.5 in the EPDS questionnaire when assessed 8 to 15 days after delivery. In the study of Vega-Dienstmaier et al. [22], no women with scores lower than 7.5 were diagnosed with DPP (sensitivity and positive predictive value of 100%). Therefore, we have decided to select patients with scores of 7.5 or higher on the EPDS scale.

### Exclusion criteria

1. Mental retardation
2. Worsening of a severe mental disorder that could hinder understanding of the objectives of the study
3. Language difficulties that impede verbal comprehension, reading and/or writing
4. Presence of a major depressive episode according to the DSM-IV-TR (depressive symptoms of sufficient intensity and duration longer than 2 weeks).

All participants will be informed that they will be randomized to one of the two study groups and will only be included if they give their informed consent to participate in the study. Women in both groups will be assessed at baseline (pre-intervention) and 6 weeks (post-intervention).

### Ethical considerations

The study will be conducted in compliance with local regulations and internationally established principles of the Declaration of Helsinki [26]. The study and protocol have been approved by the Clinical Research Ethics Committee of the Basque Country (Araba University Hospital: HS/PI2011013).

### Sample size

After a thorough review of the literature and using the Ene 2.0 [27] software, we calculated that for a statistical power of 90%, with a significance level of 5%, a sample of 30 patients per group will allow us to detect mean differences of 5.5 points on the EPDS between the experimental group and the control group (alpha = 0.05, beta = 0.10, *N* = 126). To achieve this sample, 600 participants in the third trimester of pregnancy will be screened for eligibility. Of the total, about 280 are expected to have at least one risk factor and will progress to the second phase of the study. Of these, we estimate that a quarter of the women will score 7.5 or higher on the EPDS, which would leave approximately 60 women to be randomized to the two arms of the trial.

### Randomization

All women with risk of PPD that meet the inclusion criteria and none of the exclusion criteria will be randomized with Random Allocation Software. For this purpose, all the groups from the participating hospitals will send the initials of each patient recruited to the study coordinator who will specify the allocation of the patient (to the control or intervention group); the person in charge of the assessments will be blind to this process.

### Patient assessment

Data on the wide range of clinical and demographic variables detailed below will be gathered using a data collection form. All candidate women will be assessed in the third trimester of pregnancy in order to screen for the presence of any of the aforementioned risk factors. Scales to assess socioeconomic status and personality vulnerability will also be administered in this screening. The next assessment will be the baseline assessment of all the women that are found to have at least one risk factor for PPD in the screening between 8 and 15 days after the childbirth. At this point, we will assess the mother’s physical activity and child’s temperament and measure the depressed mood of the mother with the EPDS. Women scoring ≥ 7.5 who are then then randomized to one of the two groups (control vs experimental) will be assessed weekly until the completion of six psychoeducational sessions, nearly 2 months after the baseline assessment. The control groups will be also re-assessed after the same interval.

### Demographic and other personal data

We will collect data on age, sex, ethnicity, level of education, living arrangements, employment status, problems in the delivery and medication use (in such cases).

### Risk factors

We have compiled a list of risk factors (RF) associated with PPD based on an electronic search in PubMed. The midwife will note if the pregnant women have any of these factors.

Specifically, we included the following RFs as the most closely related to PPD:

- Depressive or anxious pathology during pregnancy
- Personal history of severe mental disorder (schizophrenia and other psychoses, bipolar disorder, depressive disorder)
- Family history of severe mental disorder
- Concomitant medical diseases associated with depression (diabetes, heart disease, hypertension, obesity)
- Low or very low socioeconomic status
- Lack of support for women (partner, family, friends or others).

### Mood assessment

The **EPDS** will be used to assess participants’ depressive symptoms [22]. This 10-item self-report tool is designed to screen women for symptoms of emotional distress during pregnancy and the postnatal period.

### Vulnerable personality assessment

The Vulnerable Personality Style Questionnaire **(VPSQ)** is a nine-item self-report scale developed to assess personality traits which increase the risk of PPD [13].

### Physical Activity Questionnaires

The **IPAQ** provides a set of well-developed instruments that can be used internationally to obtain comparable estimates of physical activity. The long version we will use provides the detailed information often required in research work or for evaluation purposes [24].

The **GPAQ** was developed by WHO for monitoring physical activity [25]. It collects information about participation in physical activity and sedentary behaviour in three domains:

- Activity at work
- Activity related to traveling to and from places
- Leisure activities

### Children’s temperament style

**MP-R:** We will measure the child’s temperament with the Merrill-Palmer–Revised Scales of Development [23]. This instrument evaluates five main areas of development: cognition, language and communication, motor skills, adaptive behaviour, and socioemotional domains. It provides information about global development of the child to assess the presence of possible delays in some areas.

All the instruments to be used have demonstrated adequate psychometric properties and have been used in many other studies. Interviews will be conducted by clinicians who have good inter-rater reliability for the scales used.

### Intervention programme

Our Postpartum Depression Prevention Programme (PDPP) consists of six 1-h weekly sessions, for 2 months, focused on improving the information that the women have about depression syndrome, patient insight into their illness, early intervention for prevention, healthy lifestyles, techniques for managing anxiety, social skills and problem solving.

The first session is individual, and the remaining ones are group sessions. The programme has an open format. That is, each woman that is randomized to the experimental group will directly enter the group, and start receiving the intervention, regardless of the session number. The rationale behind this is that women should not have to wait until a group is formed. In this way, we minimize the risk of the symptoms worsening.

The programme includes the following sessions:

1. What is PPD? Etiological and trigger factors
2. Risk Factors for PPD
3. How to manage negative thoughts
4. Techniques for reducing stress and anxiety
5. Problem-solving techniques
6. Healthy lifestyle and technical guidance on seeking practical solutions

The psychoeducational sessions will only be run in Araba University Hospital. Further, the therapists will have a specific training to give the psychoeducational intervention to all women in the same way, and provide the same information, enhancing the reliability of the study.

Lastly, if participants have any question about the sessions, or about their condition, they will be able to call a telephone helpline in working hours (between 08.00 and 15.00 from Monday to Friday). The aim of this service is to facilitate participants’ access to information and address any questions they might have.

### Treatment as usual

Treatment as usual refers to the treatment that is routinely provided to pregnant women in the Spanish National Health Service. It consists of ambulatory appointments with their midwives and gynaecologist. They will not receive any therapeutic input from the psychologist.

### Expected timetable for the study

**Fig. 1:**
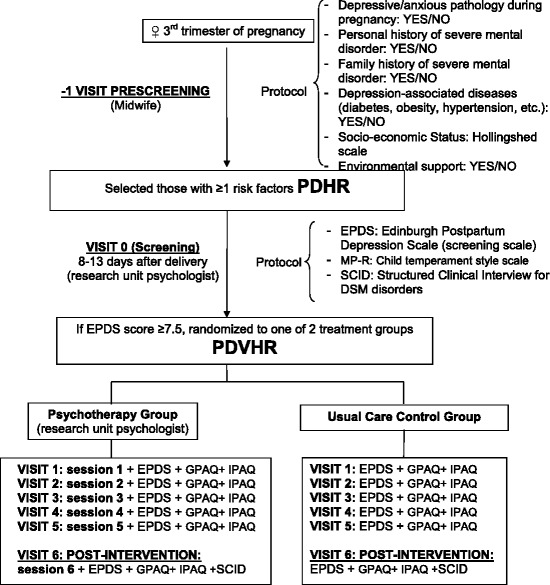
CONSORT diagram. The CONSORT diagram illustrates and reports the progression of the participants through the different points of the study

Figure 1 shows the flow of women though in the study.

### Data processing

Comparisons of baseline characteristics of the sample will be made with χ2-tests for categorical variables and the Student’s t-test or Mann Whitney U test for quantitative variables, depending on the distribution of the data. Differences in mood symptomatology during treatment will be examined using Wilcoxon signed-rank test for the within-group analyses and the Mann–Whitney U test for the between group analyses. The latter will also be used to analyse variation in EPDS scores between groups. On the other hand, cut-off points for the EPDS will be established and differences in the categorical variables created will be analysed with χ2-tests. Linear regression models will be performed for each group to evaluate the influence of the child’s temperament and global and daily activity during treatment on mood symptoms.

Univariate analyses of covariance will be conducted to investigate differences between groups. For this, the independent variable will be the group assignment (treatment as usual plus psychoeducation treatment versus treatment as usual alone) and the dependent variables will be the respective clinical or functional outcome scores post-treatment. This analysis will be performed on an intention-to-treat basis, with women being analysed in the treatment group to which they were randomized. In addition, a logistic regression model will be used to assess the relationships between clinical variables.

Finally, we will also build an ordinal regression model to explore factors associated with having depression after the intervention. The categorical variable of the EPDS assessed post-intervention will be used as the dependent variable, while risk factors evaluated at baseline will be the independent variables.

The outcomes will be reported with 95% confidence intervals. All data will be analysed using IBM SPSS Statistics v22.

## Discussion

This study will provide information about the effectiveness of a new psychoeducational programme specifically designed to prevent PPD in at-risk women. Should it be found to be effective, this programme could have an important role in preventing this type of depression, a severe mental illness, and its deleterious consequences both in women and in their family including the newborn infant.

The strengths of the study include targeting a defined sample of pregnant women at risk of depressive disorder according to the literature reviewed. The implementation of a preventive programme focused on the mental health of women during pregnancy and the postpartum period is, in itself, an important aspect of the study, as existing postpartum care programmes tend to be related to the baby and the reproductive system of the woman, as well as the treatment of health problems, but not to prevention. With this study, we underline that it is essential to pay close attention to the overall wellbeing of women including their mental health, especially in women with recognized risk factors.

## Trial status

Recruitment started on 8 September 2015, and the first participant consented on 10 September 2015. At the time of preparing this manuscript, 59 people have given consent. Recruitment is due to finish at the end of August 2017.

## Abbreviations

CBT: Cognitive Behavior therapy
EPDS: Edinburgh Postnatal Depression Scale
GPAQ: The Global Physical Activity Questionnaire
IPAQ: The International Physical Activity Questionnaire
MP-R: Merrill-Palmer-Revised Scales of Development
PDHR: Postpartum Depression High Risk
PDPP: Postpartum Depression Prevention Programme
PDVHR: Postpartum Depression Very High Risk
RF: Risk Factor
SE: Socio Economic
TAU: Treatment As Usual
VPSQ: The Vulnerable Personality Style Questionnaire

## References

[1] mdg-report-2013-english.pdf [Internet]. [cited 2016 Mar 15]. Available from: http://www.unfpa.org/sites/default/files/pub-pdf/mdg-report-2013-english.pdf. Accessed 2016.

[2] American Psychiatric Association. Diagnostic and Statistical Manual of Mental Disorders [Internet]. Fifth Edition. American Psychiatric Association; 2013 [cited 2016 Mar 15]. Available from: http://psychiatryonline.org/doi/book/10.1176/appi.books.9780890425596. Accessed 2016.

[3] Pearson RM, Evans J, Kounali D, Lewis G, Heron J, Ramchandani PG (2013). Maternal depression during pregnancy and the postnatal period: risks and possible mechanisms for offspring depression at age 18 years. JAMA Psychiat.

[4] Figueiredo FP, Parada AP, de Araujo LF, Silva WA, Del Ben CM (2014). The Influence of genetic factors on peripartum depression: A systematic review. J Affect Disord.

[5] Hoertel N, López S, Peyre H, Wall MM, González-Pinto A, Limosin F (2015). Are symptom features of depression during pregnancy, the postpartum period and outside the peripartum period distinct? Results from a nationally representative sample using item response theory (IRT). Depress Anxiety.

[6] Babb JA, Deligiannidis KM, Murgatroyd CA, Nephew BC (2015). Peripartum depression and anxiety as an integrative cross domain target for psychiatric preventative measures. Behav Brain Res.

[7] Kingston D, Janes-Kelley S, Tyrrell J, Clark L, Hamza D, Holmes P (2015). An integrated web-based mental health intervention of assessment-referral-care to reduce stress, anxiety, and depression in hospitalized pregnant women with medically high-risk pregnancies: a feasibility study protocol of hospital-based implementation. JMIR Res Protoc.

[8] Werner E, Miller M, Osborne LM, Kuzava S, Monk C (2015). Preventing postpartum depression: review and recommendations. Arch Womens Ment Health.

[9] O’Connor E, Rossom RC, Henninger M, Groom HC, Burda BU (2016). Primary care screening for and treatment of depression in pregnant and postpartum women: evidence report and systematic review for the us preventive services task force. JAMA.

[10] Pedersen C, Leserman J, Garcia N, Stansbury M, Meltzer-Brody S, Johnson J (2016). Late pregnancy thyroid-binding globulin predicts perinatal depression. Psychoneuroendocrinology.

[11] Chojenta CL, Lucke JC, Forder PM, Loxton DJ (2016). Maternal health factors as risks for postnatal depression: a prospective longitudinal study. PLoS ONE.

[12] Di Florio A, Forty L, Gordon-Smith K, Heron J, Jones L, Craddock N (2013). Perinatal episodes across the mood disorder spectrum. JAMA Psychiatry.

[13] Gelabert E, Subirà S, Plaza A, Torres A, Navarro P, Imaz ML (2011). The Vulnerable Personality Style Questionnaire: psychometric properties in Spanish postpartum women. Arch Womens Ment Health.

[14] Bobes Bascarán MT, Jover M, Llácer B, Carot JM, Sanjuan J (2011). Spanish adaptation of the EAS Temperament Survey for the assessment of child temperament. Psicothema.

[15] Alasoom LI, Koura MR (2014). Predictors of postpartum depression in the eastern province capital of Saudi Arabia. J Fam Med Prim Care.

[16] Pilkington PD, Milne LC, Cairns KE, Lewis J, Whelan TA (2015). Modifiable partner factors associated with perinatal depression and anxiety: a systematic review and meta-analysis. J Affect Disord.

[17] Biaggi A, Conroy S, Pawlby S, Pariante CM (2016). Identifying the women at risk of antenatal anxiety and depression: A systematic review. J Affect Disord.

[18] Blackmore ER, Côté-Arsenault D, Tang W, Glover V, Evans J, Golding J (2011). Previous prenatal loss as a predictor of perinatal depression and anxiety. Br J Psychiatry J Ment Sci.

[19] Goyal D, Gay C, Lee KA (2010). How much does low socioeconomic status increase the risk of prenatal and postpartum depressive symptoms in first-time mothers? Womens Health Issues Off. Publ Jacobs Inst Womens Health.

[20] Cuijpers P, Andersson G, Donker T, van Straten A (2011). Psychological treatment of depression: results of a series of meta-analyses. Nord J Psychiatry.

[21] Brugha TS, Morrell CJ, Slade P, Walters SJ (2011). Universal prevention of depression in women postnatally: cluster randomized trial evidence in primary care. Psychol Med.

[22] Vega-Dienstmaier JM, Mazzotti Suárez G, Campos Sánchez M (2002). Validation of a Spanish version of the Edinburgh postnatal depression scale. Actas Esp Psiquiatr.

[23] Sánchez Sánchez, Fernando, Fernandez Santamaria, Pablo, Fernández-Pinto, Irene, Arribas Águila,David. Merrill-Palmer-R Escalas de desarrollo [Internet]. Madrid: TEA Ediciones S.A.; 2011. Available from: http://www.web.teaediciones.com/Ejemplos/Extracto_Manual_MPR_web.pdf

[24] Martínez-González MA, López-Fontana C, Varo JJ, Sánchez-Villegas A, Martinez JA (2005). Validation of the Spanish version of the physical activity questionnaire used in the Nurses’ Health Study and the Health Professionals’ Follow-up Study. Public Health Nutr.

[25] World Health Organization. Global Physical Activity Questionnaire (GPAQ) [Internet]. Geneva; Available from: http://www.who.int/chp/steps/GPAQ/en/. Accessed 2016.

[26] World Medical Association (2013). World Medical Association Declaration of Helsinki: Ethical principles for medical research involving human subjects. JAMA.

[27] Busquets LB, Marino AP. Cálculo del tamaño muestral (TM) con el programa Ene 2.0: manual del programa, documentación y ejemplos [Internet]. Gráficas Monterreina; 2005. Available from: https://books.google.es/books?id=1j1INQAACAAJ. Accessed 2016.

